# The Effect of the COVID-19 Pandemic on the Characteristics of Inpatients at the Orthopaedics and Traumatology Clinic in a Secondary Care Public Hospital in Turkey

**DOI:** 10.7759/cureus.34706

**Published:** 2023-02-06

**Authors:** İBRAHİM EKE

**Affiliations:** 1 Orthopedics and Traumatology, Antalya Atatürk State Hospital, Antalya, TUR

**Keywords:** trauma surgeries, orthopaedic surgeries, emergency surgeries, elective surgeries, covid -19 pandemic

## Abstract

Objective

The aim of this study was to evaluate patients who were hospitalized at an orthopaedics and traumatology clinic in a secondary care public hospital in Turkey during the first two years of the COVID-19 pandemic.

Methods

This was a cross-sectional and retrospective study that included a total of 7439 patients - those who had been hospitalized between 11 March 2020, the date of the first confirmed case of COVID-19 in Turkey, and 10 March 2022 (2949 patients), and those hospitalized in the same clinic between 11 March 2018 and 10 March 2020, designated as the pre-pandemic group (control group; 4490 patients). Patients were divided into three groups: <18 years old, 18-65 years old and >65 years old and compared separately in terms of clinical, diagnostic and therapeutic characteristics as pandemic patients and control group.

Results

Despite the decline in both the number of admissions to the emergency service and visits to the outpatient clinic among inpatients during the pandemic period, the rate of admissions to the emergency department remained higher than that of the control group throughout the pandemic period (p<0.001). Surgical procedures were lower both numerically and proportionally in the patients who presented during the pandemic than in the pre-pandemic period (p<0.001). While the rate of traumatic surgery was higher in the pandemic group (29%) than in the pre-pandemic group (26.7%), the rate of elective surgery was higher in the pre-pandemic group (71.3%) than in the pandemic one (67.5%) (p=0.037 and p=0.001).The number of patients with fractures in all age groups decreased numerically in the pandemic cohort. While no significant difference was observed between pandemic and pre-pandemic cohorts in terms of the length of hospitalization in all age groups, Intensive Care Unit (ICU) hospitalization rate was found to have increased significantly in adult and elderly patient groups during the pandemic (p<0.001).

Conclusion

In our study, when the number of patients who underwent orthopaedic surgical treatment, in general, was examined, it appeared that the number of both traumatic and elective surgeries decreased during the pandemic significantly. It was found that the ICU stay rate increased significantly in adult and elderly patient groups during the pandemic. Although there were no confirmed cases of COVID-19 among the patients included in the study, it is known that the pandemic and especially the lockdown periods adversely affected the mental, physical and biological health of individuals.

In this context our study will be able to serve as a guide for taking measures like: 1. increasing the ICU capacity of hospitals, 2. providing in-service training to improve the experience of nurses, doctors or other healthcare workers, especially in specialized units such as ICUs, operating rooms and emergency services, considering the number of personnel who may be affected by the pandemic, and 3. ensuring a balanced distribution of orthopaedic operations in private and public hospitals, to reduce the negative effects on orthopaedic health services of other pandemics that may arise in the future.

## Introduction

The COVID-19 pandemic progressed with extremely high morbidity and mortality rates before the start of vaccination among all ages, genders, and races, especially in patients with advanced age and comorbidities. Especially due to the restriction of physical movement, the periods of lockdowns caused an obvious decline in the number of trauma cases, and it turned out that emergency service visits as a result of traffic accidents, falls, and assaults decreased considerably [1,2]. In the process, significant changes were observed in the way people sought health care and treatment. Research has even shown that mortality rates increased due to myocardial infarction during the pandemic, notably because of refusal to present to the hospital [3,4]. Similar conditions appeared for both elective and emergency cases of orthopaedics and traumatology [5].

There are many studies being conducted by a number of universities and public hospitals in many countries, investigating the effects of the COVID-19 pandemic on the working conditions of orthopaedics and traumatology clinics, residency training programs, and related surgeries [5-7]. After the first confirmed case of COVID-19 in Turkey on 11 March 2020, elective surgeries were halted in all public hospitals across the country for a period of 4-6 months. Although some elective surgery cases were performed from time to time in periods when the number of COVID-19 cases was relatively lower, and especially after large-scale vaccinations in the second year of the pandemic, the surgery schedule has not been the same as that of the pre-pandemic period in our clinic in this two-year period. Especially during periods when the number of COVID-19 cases increased, it was observed that there was a decline in orthopaedics and trauma cases presented to the emergency service as well as the orthopaedics and traumatology outpatient clinics of our hospital. The data obtained from our study, with the hypothesis that the number of orthopaedics and traumatology emergency cases and elective surgery/conservative treatments declined during the COVID-19 pandemic (which has completed two years in our country as of March 2022), is expected to act like a guideline in terms of effective healthcare planning in case of pandemics that may be experienced again in the future.

The aim of this study was to evaluate the patients hospitalized at the orthopaedics and traumatology clinic in a secondary care public hospital during the first two years of the pandemic in Turkey; compare the demographic, clinical, diagnosis and treatment characteristics of pre-pandemic patients by different age groups; and investigate the impact of the pandemic on the rate of orthopaedic emergency cases, elective surgeries, and conservative treatments.

## Materials and methods

Study design and participants

This cross-sectional and retrospective study was not conducted with randomization, and included all patients who underwent elective or emergency surgery (both orthopaedic or trauma) and/or had conservative treatment after being hospitalized at the Orthopaedics and Traumatology Clinic in a secondary care public hospital in Turkey for two different two-year periods - between 11 March 2020, the date of the first confirmed case of COVID-19 in our country, and 10 March 2022 (2949 patients), and between 11 March 2018 and 10 March 2020 (pre-pandemic or control group; 4490 patients). The population of the study consisted of 7592 patients who were hospitalized and treated in those two periods. One hundred and fifty-three patients with missing or conflicting data regarding age, diagnosis or treatment were excluded from the study, which then consisted of a total of 7439 patients.

During the research process at our hospital, pre-operative polymerase chain reaction (PCR) tests were performed on all patients who were planned for elective surgery, and patients with COVID-positive tests were not hospitalized and their surgeries were postponed. If these patients were symptomatic for COVID-19 and needed to be treated and followed up at the hospital, they were hospitalized at the pandemic services, not at the orthopaedics and traumatology service. On the other hand, COVID-positive cases who underwent traumatic surgery were similarly treated and followed up at the pandemic services or, if needed, at the pandemic ICU. The patients followed and treated at the orthopaedics and traumatology service included in this study were COVID-negative.

Data collection

The data of the patients were obtained from the electronic patient files obtained from the Hospital Data Processing Unit. Necessary information such as age, gender, anatomical regions/tissues affected by the disease (upper extremity bones/lower extremity bones/axial skeleton bones/soft tissue), the first point of clinical presentation of the patients (orthopaedic outpatient clinic or emergency department), the treatment applied (conservative/surgery-trauma surgery/elective surgery), the length of hospital stay and the Intensive Care Unit (ICU) hospitalization, if any, were recorded in data collection forms. No specific age range was determined for the patients included in the study, with the patients being divided into three groups: paediatric (<18 years old), adults (18-65 years old), and the elderly (>65 years old). Patients before and during the pandemic were compared separately by age groups in terms of demographic, clinical, diagnostic, and treatment characteristics.

Ethical approval

Prior to the study, necessary ethical approval was obtained from the Clinical Research Ethics Committee of the University of Health Sciences, Antalya Training and Research Hospital (Date: 30.06.2022, decision number: 13/5). The study was conducted in accordance with the Declaration of Helsinki.

Statistical analysis

The Shapiro-Wilk test was used to test the normality of the data. Continuous variables were given with their mean, standard deviation (SD), median, and range (min-max). Categorical variables were presented with frequencies (n) and percentages (%). Pearson chi-square test and Fisher’s exact test were used to determine the relationship between categorical variables. The Mann-Whitney U test and Independent t-test were used for comparisons of continuous data between two independent groups. Statistical analysis was performed using IBM SPSS Statistics for Windows, Version 23.0 (IBM Corp., Armonk, USA). A value of two-sided p<0.05 was considered statistically significant.

## Results

The median age was calculated as 59 in both the pre-pandemic and pandemic cohorts. It appeared that 59.5% of the patients in the control group and 56.6% of the pandemic cohort were female. Admission with lower extremity pathologies was statistically significantly higher in the control group (68.8%), and admission with soft tissue pathologies was statistically significantly higher in the pandemic group (8.1%) (all p<0.001). It appears that there was a decline in both the number of admissions to the emergency service and outpatient clinic during the pandemic, compared to the pre-pandemic period, and that the rate of admissions to the outpatient clinic (64.5%) was lower than that of the control group (71%) during the pandemic, along with the rate of admission to emergency service being higher than the control group (p<0.001). Surgical treatment procedures performed were lower in the pandemic cohort both numerically (2844) and proportionally (96.4%) (p<0.001); the rate of traumatic surgery cases (29%) was higher in the pandemic cohort and the rate of elective surgery was found higher in the pre-pandemic group (71.3%) (p=0.037 and p=0.001, respectively) (Table 1).

**Table 1:** Demographics and first admission to hospital and choice of treatment data for the control and pandemic cohort Mann-Whitney U test, Pearson chi-square test. Since there are more than one affected anatomical region/tissue and/or more than one treatment option (both conservative treatment and surgical treatment) in some of the patients, the sum of the rates may not be 100%.

Variables	Control (n=4490)	Pandemic (n=2949)	p-value
Age (years), median(range)	59 (2-99)	59 (2-95)	0.061
Female	64 (2-97)	63 (2-95)	0.100
Male	46 (3-99)	47 (2-91)	0.726
Gender, n(%)			
Female	2673 (59.5)	1670 (56.6)	0.013
Male	1817 (40.5)	1279 (43.4)	
Affected anatomical regions and tissues, n(%)			
Upper extremity bones	1248 (27.8)	822 (27.9)	0.941
Lower extremity bones	3087 (68.8)	1891 (64.1)	<0.001
Axial skeleton bones	30 (0.7)	20 (0.7)	0.959
Soft tissues	146 (3.3)	238 (8.1)	<0.001
First admission to, n(%)			
Emergency Service	1303 (29)	1046 (35.5)	<0.001
Orthopaedic outpatient clinic	3187 (71)	1903 (64.5)	
Choice of treatment, n(%)			
Conservative treatment	159 (3.5)	120 (4.1)	0.241
Surgical treatment	4400 (98)	2844 (96.4)	<0.001
Traumatic	1201 (26.7)	854 (29)	0.037
Elective	3199 (71.3)	1990 (67.5)	0.001

On evaluating patients by dividing them into three age groups, no statistically significant difference was found in the mean age of patients treated before and during the pandemic in all three age groups (p=0.776, p=0.642, p=0.155). As regards to the affected anatomical region, pathologies related to lower extremities were found to be statistically significant in the 18-65 age group in the pre-pandemic period (63.6%) (p=0.003), while pathologies related to soft tissues were statistically significant in all three age groups during the pandemic period (24.1%, 8.4%, 4.8%) (p=0.001, p<0.001, p=0.009, respectively, by age group). Despite the decreased number of cases with fractures in all age groups in numerical terms, there was an increase in the rate of cases with short bone fractures (14%) (p=0.025) in the 18-65 age group and in the rate of cases with long bone fractures in elderly patients (11.6%) (p=0.002) during the pandemic period. On the other hand, a significant decrease in the rate of cases with dislocation was detected in the 18-65 age group during the pandemic period (0.9%) (p=0.002). Table 2 presents the details of emergency service and outpatient clinic admissions together with the diagnoses (Table 2).

**Table 2:** Comparison of demographic characteristics, diagnosis and treatment choices for the control and pandemic cohort according to age groups Independent t-test, Mann-Whitney U test, Pearson chi-square test, Fisher’s exact test. Since there are more than one affected anatomical region/tissue and/or more than one fracture in some of the patients, the sum of the rates may not be 100%.

	<18 years			18-65 years			>65 years		
Variables	Control (n=144)	Pandemic (n=158)	p-value	Control (n=2814)	Pandemic (n=1835)	p-value	Control (n=1532)	Pandemic (n=956)	p-value
Age (years), mean±SD	12.6±4.1	12.5±4.4	0.776	47.5±13.6	47.3±14.1	0.642	73.2±6.1	73.5±6	0.155
Gender, n(%)									
Female	33 (22.9)	30 (19)	0.401	1448 (51.5)	948 (51.7)	0.891	1192 (77.8)	692 (72.4)	0.002
Male	111 (77.1)	128 (81)		1366 (48.5)	887 (48.3)		340 (22.2)	264 (27.6)	
Affected anatomical regions and tissues, n(%)									
Upper extremity bones	87 (60.4)	79 (50)	0.069	927 (32.9)	591 (32.2)	0.601	234 (15.3)	152 (15.9)	0.675
Lower extremity bones	41 (28.5)	44 (27.8)	0.904	1790 (63.6)	1088 (59.3)	0.003	1256 (82)	759 (79.4)	0.109
Axial skeleton bones	3 (2.1)	0(0)	0.107	24 (0.9)	16 (0.9)	0.945	3 (0.2)	4 (0.4)	0.439
Soft tissues	14 (9.7)	38 (24.1)	0.001	89 (3.2)	154 (8.4)	<0.001	43 (2.8)	46 (4.8)	0.009
First admission to, n(%)									
Emergency Service	112 (77.8)	124 (78.5)	0.883	881 (31.3)	685 (37.3)	<0.001	310 (20.2)	237 (24.8)	0.008
Orthopaedic outpatient clinic	32 (22.2)	34 (21.5)		1933 (68.7)	1150 (62.7)		1222 (79.8)	719 (75.2)	
Emergency Service, n(%)									
Fractures	112 (77.8)	110 (69.6)	0.109	863 (30.7)	614 (33.5)	0.046	308 (20.1)	223 (23.3)	0.056
Long bone fractures	88 (61.1)	81 (51.3)	0.085	450 (16)	315 (17.2)	0.291	120 (7.8)	111 (11.6)	0.002
Flat bone fractures	3 (2.1)	0 (0)	0.107	42 (1.5)	28 (1.5)	0.927	6 (0.4)	4 (0.4)	0.999
Short bone fractures	21 (14.6)	27 (17.1)	0.552	331 (11.8)	257 (14)	0.025	26 (1.7)	21 (2.2)	0.373
Femoral neck fractures	0 (0)	2 (1.3)	0.499	52 (1.8)	28 (1.5)	0.409	156 (10.2)	87 (9.1)	0.376
Dislocations	0 (0)	1 (0.6)	0.999	57 (2)	16 (0.9)	0.002	20 (1.3)	11 (1.2)	0.735
Orthopaedic outpatient clinic, n(%)									
Gonarthrosis	0 (0)	0 (0)	-	597 (21.2)	323 (17.6)	0.003	897 (58.6)	494 (51.7)	0.001
Coxarthrosis	0 (0)	0 (0)	-	99 (3.5)	44 (2.4)	0.031	64 (4.2)	9 (0.9)	<0.001
Rotator cuff pathology	3 (2.1)	1 (0.6)	0.351	521 (18.5)	279 (15.2)	0.003	174 (11.4)	97 (10.1)	0.346
Meniscopathy	1 (0.7)	4 (2.5)	0.374	459 (16.3)	212 (11.6)	<0.001	10 (0.7)	9 (0.9)	0.421
Hallux valgus	7 (4.9)	8 (5.1)	0.936	184 (6.5)	189 (10.3)	<0.001	21 (1.4)	39 (4.1)	<0.001
Soft tissue pathology	21 (14.6)	37 (23.4)	0.052	81 (2.9)	151 (8.2)	<0.001	46 (3)	42 (4.4)	0.068
Other pathologies	0 (0)	0 (0)	-	10 (0.4)	33 (1.8)	<0.001	9 (0.6)	40 (4.2)	<0.001

As can be seen in Table 3, treatment-related processes such as treatment applied in three different age groups, lengths of hospital stay and ICU hospitalization were evaluated by comparing them in relation to pre-pandemic and pandemic periods. During the pandemic, all orthopaedic surgical treatment applications in the 18-65 and >65 aged patient groups decreased statistically significantly, both numerically (1760 and 935, respectively) and proportionally (95.9% and 97.8)%, respectively) (p<0.001, p=0.021, respectively, by age group). While no significant difference was observed between the pandemic and pre-pandemic groups in terms of length of hospital stay among all age groups, the ICU stay rate appeared to have increased significantly in the adult (11.3%) and elderly patient groups (16.8%) during the pandemic (all p<0.001) (Table 3).

**Table 3:** Comparison of treatment choices, hospitalization days and ICU hospitalization status for the control and pandemic cohort according to age groups Mann-Whitney U test, Pearson chi-square test, Fisher’s exact test. FNF: Femur neck fractures, ICU: Intensive care unit Since there are more than one treatment option (both conservative treatment and surgical treatment), conservative treatment option (both conservative treatment of fractures and closed reduction of dislocation) in some of the patients, the sum of the rates may not be 100%.

	<18 years			18-65 years			>65 years		
Variables	Control (n=144)	Pandemic (n=158)	p-value	Control (n=2814)	Pandemic (n=1835)	p-value	Control (n=1532)	Pandemic (n=956)	p-value
Choice of treatment, n(%)									
Conservative treatment	10 (6.9)	10 (6.3)	0.830	117 (4.2)	84 (4.6)	0.491	32 (2.1)	26 (2.7)	0.310
Surgical treatment	134 (93.1)	149 (94.3)	0.655	2750 (97.7)	1760 (95.9)	<0.001	1516 (99)	935 (97.8)	0.021
Conservative treatment, n(%)									
Conservative treatment of fractures	10 (6.9)	9 (5.7)	0.655	64 (2.3)	74 (4)	0.001	14 (0.9)	18 (1.9)	0.037
Closed reduction of dislocation	0 (0)	1 (0.6)	0.999	57 (2)	16 (0.9)	0.002	20 (1.3)	11 (1.2)	0.735
Surgical treatment, n(%)									
Traumatic surgery	102 (70.8)	101 (63.9)	0.201	803 (28.5)	545 (29.7)	0.392	296 (19.3)	208 (21.8)	0.141
Elective surgery	32 (22.2)	48 (30.4)	0.109	1947 (69.2)	1215 (66.2)	0.033	1220(79.6)	727 (76)	0.035
Traumatic surgery, n(%)									
Arthroplasty (in patients diagnosed with FNF)	0 (0)	0 (0)	-	49 (1.7)	28 (1.5)	0.574	153 (10)	87 (9.1)	0.466
Surgical treatment of fractures	102 (70.8)	101 (63.9)	0.201	754 (26.8)	517 (28.2)	0.302	143 (9.3)	121 (12.7)	0.009
Elective surgery, n(%)									
Arthroplasty	0 (0)	0 (0)	-	696 (24.7)	367 (20)	<0.001	961 (62.7)	503 (52.6)	<0.001
Arthroscopy	1 (0.7)	4 (2.5)	0.374	456 (16.2)	212 (11.6)	<0.001	10 (0.7)	9 (0.9)	0.421
Rotator cuff repair	3 (2.1)	1 (0.6)	0.351	520 (18.5)	273 (14.9)	0.001	173 (11.3)	96 (10)	0.329
Hallux valgus surgery	7 (4.9)	8 (5.1)	0.936	184 (6.5)	189 (10.3)	<0.001	21 (1.4)	39 (4.1)	<0.001
Soft tissue surgery	21 (14.6)	35 (22.2)	0.091	81 (2.9)	145 (7.9)	<0.001	46 (3)	42 (4.4)	0.068
Other surgical procedures	0 (0)	0 (0)	-	10 (0.4)	29 (1.6)	<0.001	9 (0.6)	38 (4)	<0.001
Hospitalization days, median(range)	2 (1-17)	2 (1-18)	0.763	3 (1-21)	3 (1-38)	0.337	5 (1-50)	5 (1-50)	0.190
ICU hospitalization, n(%)	16 (11.1)	11 (7)	0.207	218 (7.7)	207 (11.3)	<0.001	146 (9.5)	161 (16.8)	<0.001
ICU hospitalization days, median(range)	1 (1-5)	1 (1-3)	0.716	1 (0-11)	1 (0-8)	0.715	1 (0-5)	1 (0-3)	0.515

## Discussion

In this sudy, it was aimed to evaluate the general demographic, clinical and treatment characteristics of the patients who were hospitalized and treated in the first two years of the COVID-19 pandemic at the orthopaedics and traumatology clinic in a secondary care public hospital in Turkey. Although there was a decrease in the number of admissions to the emergency department and the orthopaedic outpatient clinic in inpatients during the pandemic period, the rate of admission to the emergency department during the pandemic period was found to be higher than the control group. Surgical procedures were lower both numerically and proportionally in the group of patients who presented during the pandemic, with the rate of emergency surgery being higher in the same group, while the rate of elective surgery was higher in the pre-pandemic group. The number of patients with fractures in all age groups decreased numerically in the pandemic cohort. While no significant difference was observed between the pandemic and pre-pandemic cohorts in terms of the length of hospitalization among all age groups, the ICU hospitalization rate was found to have increased significantly in the adult and elderly patient groups during the pandemic.

As already mentioned, there are various studies investigating the effects of the COVID-19 pandemic on orthopaedics and traumatology residency training. One of those studies investigated the impact of the COVID-19 pandemic on orthopaedic surgery residency training in the United States and reported that the pandemic caused significant changes in the training experience of orthopaedic surgery residents, remarkably reduced clinical working hours and case volume, and led to worsening in such training [8]. A similar study conducted in India concluded that the COVID-19 pandemic adversely affected the academic training of residents, their surgical exposure and hands-on training [9]. Although our hospital does not provide resident training due to being a secondary care public hospital, we believe that the pandemic has had negative effects not only on the training of orthopaedics and traumatology residents but also on the surgical practices of orthopaedics and traumatology specialists, since frequent elective surgery cases were halted, especially in public hospitals.

After the first confirmed case of COVID-19 in Turkey on 11 March 2020, elective surgeries were halted in all public hospitals across the country. It was apparent that there was a significant decrease in orthopaedics and trauma cases admitted to the emergency department as well as to the orthopaedics and traumatology outpatient clinics of our hospital during the waves and lockdowns, when the number of COVID-19 cases increased. In the literature, similar studies have been found, especially in the early months of the pandemic, in support of the results of our study based on the hypothesis that the number of orthopaedics and traumatology emergency and elective surgery/conservative treatments decreased during this period. For example, a systematic review of 36 original articles reported that orthopaedic and trauma surgery was clearly affected by the pandemic. The same study indicated that the number of elective visits declined by 50.0% to 74.0%, the number of emergency and trauma visits showed a decrease by 37.7% to 74.2%, the number of trauma surgery decreased by 21.2% to 66.7%, and that of elective surgeries by 33.3% to 100%. Furthermore, the study also emphasized the importance of continuing with the treatment and surgical care in order to not adversely affect the treatment progress of the patients [10]. Another study conducted in Chile showed that there was a significant decrease in the number of orthopaedic procedures due to the COVID-19 pandemic throughout 2020. The same study also reported that the pandemic had a significantly negative impact on the public health network compared to the private health institutions in the performance of elective surgeries. In addition, the study mentioned that such a situation may result in a long waiting period for patients to undergo orthopaedic surgery [11]. Another study conducted in Czechia showed that there was a significant decrease in healthcare services for orthopaedics and trauma patients due to the COVID-19 pandemic. In the same study, three different quarantine periods were compared with the same periods of three years before the pandemic, and the rates of orthopaedic outpatient clinic admissions were found to have decreased by 54.12%, 42.88% and 34.53%, in the first, second, and third lockdowns, respectively, and likewise, the number of elective surgeries had decreased by 69.01%, 87.57% and 74.89%, and the number of emergency surgeries by 33.15%, 37.46% and 27.24%, respectively [12]. In our study, the number of patients who were admitted to the outpatient clinic during the pandemic was found to have decreased by 40.29%, the number of patients admitted to the emergency service by 19.72%, and the number of patients undergoing operations by 35.37% (emergency surgeries decreased by 28.90% and elective surgeries by 37.72%). In line with the results of our study, the decrease in the number of orthopaedic and trauma patient admissions and surgeries during the pandemic period was lower than that reported in the literature, which may be related to the fact that those studies were conducted in the early period when the pandemic became more severe and lockdowns were being implemented.

With two research designs being most similar to ours, the study conducted by Kalem et al., investigating the effects of the COVID-19 pandemic curfew on orthopaedic trauma in our country, reported that the number of admissions due to trauma decreased by 50.9% during the pandemic, but the rate of trauma throughout the skeletal system, especially in the upper extremity, was reported to have increased from 30.5% to 49.9% [5]. The second study similar to ours, conducted by Turgut et al. reported that, compared to the pre-pandemic period, the frequency of fractures decreased by about one-third during the pandemic, the average age of the patients with fractures in the paediatric group decreased, and finger fractures in paediatric patients and metatarsal fractures in adult patients decreased significantly during the pandemic [13].

In our study, trauma admissions were found to have decreased by 27.92% and the frequency of fractures by 25.79%. No significant change was observed in the mean age of patients in the pre-pandemic and pandemic periods, not only in paediatric patients but also in adult and elderly patient groups. Unlike the study of Turgut et al., our study found an increase in the rate of short bone fractures in adult patients and long bone fractures in elderly patients during the pandemic period.

We have previously mentioned that admissions due to soft tissue pathologies and soft tissue surgery increased accordingly, during the pandemic. This could be explained by the fact that the number of elective surgeries, such as knee/hip arthroplasty decreased due to the restrictions on the use of orthopaedic medical equipment in public hospitals during the pandemic, resulting in the concentration of orthopaedic surgeons in this area. Or it can be explained by the fact that the patients had the chance to have some soft tissue pathology surgeries/treatments that they postponed, thanks to their relatively flexible working hours.

The pandemic seems to have caused a decrease in the number of elective operations not only in orthopaedics and traumatology but also in all surgical branches. For example, the study by Ode et al. in Nigeria reported that there was a significant decrease in elective surgical services provided at the hospital during the first wave of the COVID-19 pandemic compared to the corresponding period in the previous year. It was also reported that immediate measures should be taken in that regard since a decline in elective surgery services would negatively affect access to healthcare services apart from the excessive accumulation of elective surgical procedures [14]. On the other hand, a literature review conducted by Phillips et al., which included 11 reports from nine different health organizations, stated that with the global guidance of major medical associations, they agreed that elective surgical procedures should be postponed in order to minimize the risk of COVID-19 spread and to increase available hospital resources to manage the flow of COVID-19 patients. The study also stressed the need for clinicians and patients to evaluate conservative treatment options until surgical treatment options became available again, and to manage increased surgical waitlists caused by the elective surgical shutdown [15]. It was exactly the case in our clinic in line with the recommendations and guidelines of the Turkish Ministry of Health, since elective surgical procedures had been postponed, especially during the periods when the number of COVID-19 cases increased. In the process, elective surgical procedures in our country were executed in private hospitals where COVID-19 cases were followed and treated relatively less than they were in public hospitals, preventing the increase in orthopaedic surgery and other surgical workload and so, the crisis was well managed. This can be shown as a situation that alone can explain the decrease in the number of orthopaedic cases compared to the pre-pandemic period. However, the fact that private hospitals are not accessible to patients of all income groups may have caused inequality in the provision of health services. On the other hand, the closure of schools, being/working at home in a safer and more protected environment during the pandemic and preventing people from travelling during the lockdown periods also helped people living in a safer environment led to a decrease in especially trauma cases.

Our study differs from the studies in the literature since it evaluated the first two years in which the pandemic was largely effective. This could be the strength of our study. Another strong aspect could be that approximately 7500 pandemic and pre-pandemic patients who were hospitalized and treated with both trauma-orthopaedic and traumatic-elective clinic applications were evaluated.

The fact that our study was single-centred can be a limitation and the evaluation of the data to be obtained in similar secondary and even tertiary care hospitals would have made our study stronger. Since it is a secondary care public hospital, there are no trauma/hand/spine surgeons or musculoskeletal oncologists. So special cases are generally referred to a tertiary advanced center. Another limitation of our study is that more detailed information about the patients could not be reached since the data of approximately 7500 patients were obtained from the hospital automation system.

## Conclusions

In our study, when the number of patients who underwent orthopaedic surgical treatment in general, was examined, it appeared that the number of both traumatic and elective surgeries decreased during the pandemic, significantly. While no significant difference was observed between the pandemic and pre-pandemic groups in terms of the length of hospital stay among all age groups, it was found that the ICU stay rate increased significantly in adult and elderly patient groups during the pandemic. Although there were no confirmed cases of COVID-19 among the patients included in the study, it is known that the pandemic and especially the lockdown periods adversely affected the mental, physical and biological health of individuals.

In this context, our study will be able to serve as a guide for taking measures like: 1. increasing the ICU capacity of hospitals, 2. providing in-service training to improve the experience of nurses, doctors or other healthcare workers, especially in specialized units such as ICU, operating rooms and emergency services, considering the number of personnel who may be affected by the pandemic, and 3. ensuring a balanced distribution of orthopaedic operations in private and public hospitals, to reduce the negative effects on orthopaedic health services of other pandemics that may arise in the future .
